# Epidemiological profile of dengue in Zhejiang Province, southeast China

**DOI:** 10.1371/journal.pone.0208810

**Published:** 2018-12-11

**Authors:** Jiangping Ren, Feng Ling, Jimin Sun, Zhenyu Gong, Ying Liu, Xuguang Shi, Rong Zhang, Yujia Zhai, Enfu Chen, Zhiping Chen

**Affiliations:** 1 Zhejiang Provincial Centre for Disease Control and Prevention, Hangzhou, China; 2 Key Laboratory of Vaccine, Prevention and Control of Infectious Disease of Zhejiang Province, Hangzhou, China; 3 Field Epidemiology Training Program of Zhejiang Province, Hangzhou, China; The University of Hong Kong, CHINA

## Abstract

**Background:**

Dengue is one of the most important vector-bore infectious diseases in China because of its drastic increase in incidence, geographic extension and profound influence on China’s economy. This study aims to retrospectively uncover the epidemiological profile of dengue in Zhejiang, one of the most developed provinces in China, and to find the problem existing in dengue control and prevention.

**Methodology:**

Descriptive analyses on the dengue incidence and associated factors were performed. We also identified potential space-time cluster and generated the risk map of dengue.

**Principal findings:**

A total of 529 cases were reported in Zhejiang Province from 2005 to 2016, and 44.4% were imported. 67.7% of cases were 25~60 years old and the overall male-to-female sex ratio was 1.09:1. Dengue was reported all year round and 70.7% of cases occurred between August and October. Indigenous cases were only reported in the period between July to November and more than half occurred in September. Geographically, dengue was most distributed in Jinghua (3.62 per million), Shaoxing (1.00 per million) and Taizhou (0.81 per million) prefecture level cities. Outbreaks were confirmed in Yiwu, Keqiao and Huangyan counties in 2009, 2015, and 2016, respectively. 73.9% cases would seek medical advice within two days after onset and be confirmed within 9 days after onset. 75.6% would be recognized as dengue within 8 days after their first visit. The time intervals between onset and confirmation (median 7 vs 6 days; Wilcoxon rank sum test *Z* = -2.40, *P* = 0.016), first visit and confirmation (median 7 vs 6 days; Wilcoxon rank sum test *Z* = -2.59, *P =* 0.009) of indigenous cases were significantly longer than those of imported ones. However, the time intervals between onset and first visit for indigenous cases was shorter (median 0 vs 1 days; Wilcoxon rank sum test *Z* = -2.10, *P* = 0.036). Fever (99.1%), fatigue (81.9), rash (63.7%), headache (67.2%) and myalgia (52.60%) were the most frequently mentioned symptoms.

**Conclusions:**

Zhejiang has recently witnessed an increase in incidence and geographic extension of dengue. Timely diagnosis is important to stop local transmission and outbreak.

## Introduction

Dengue, also named as break-bone fever, is one of the most important reemerging vector-borne acute viral diseases. It is widely distributed in Southeast Asia, Africa, South America, the Western Pacific Region and the Eastern Mediterranean Region [1]. In the past 50 years, the incidence has increased 30 fold with increasing geographic expansion to new countries and from urban to rural settings [1]. Because of the non-specific and self-limited symptoms, dengue is always misclassified and underreported. It is estimated that 3.9 billion people in 128 countries are at risk of dengue virus (DENV) infection, 390 million people are attacked by DENV per year and 96 million of them manifest apparently (at any level of clinical or sub-clinical severity)[2, 3]. Another study published in 2016 indicated that there were 58.40 million symptomatic DENV infections globally in 2013, and the total annual global cost of dengue illness was US$8∙9 billion [4]. DENV is a single-stranded RNA virus and comprises four distinct but antigenic related serotypes (DENV-1, DENV-2, DENV-3 and DENV-4). It belongs to the family Flaviviridae and the genus Flavivirus. The spectrum of infection ranges from asymptomatic disease to undifferentiated fever, classical dengue fever and severe dengue fever (dengue hemorrhagic fever, dengue shocks syndrome) [1]. Aedes aegypti and aedes albopictus mosquito are the primary vectors of dengue. Other transmission routes, such as vertical transmission, blood transfusion, organ transplantation and needlestick were also reported but limited [5, 6].

The first reported dengue outbreak after 1949 in mainland China occurred in Guangdong Province in 1978. After that, dengue outbreaks have been reported in Hainan, Guangxi, Fujian, Zhejiang and Yunnan provinces. All of those provinces are in the southeast coastal regions or in the area bordering Laos and Vietnam [7, 8]. In 2013, an outbreak was reported in the central of Henan Province, which is located in the center of China and is in a warm temperate zone with a sub-humid monsoon climate [9]. From 1978 to 2014, a total of 708,073 dengue cases were reported in mainland China, resulting in 616 deaths, and all of the four serotype DENV have been detected [7]. Zhejiang Province is located in southeastern China. It is classified as one of high-risk dengue epidemic provinces in China by Chinese Center for Disease Control and Prevention (CCDC). This study uncovers the epidemiological profile and trend of dengue in Zhejiang province, and tries to explore the shortness in the disease control.

## Materials and methods

### Ethics statement

On 1 September 1989, dengue was made statutorily notifiable in China. As an important part of continuing public health surveillance, the collection and analysis of data about certain notifiable infectious disease by public health officials in charge of risk assessment and policy proposal are exempt from institutional review board assessment. All of the collected data were supplied in an anonymous format and no individual identifying information were provided. This is authorized by the Law of the People’s Republic of China on Prevention and Treatment of Infectious Diseases.

### Diagnosis and reporting

Dengue cases were defined according to *the diagnostic criteria and principles of management for dengue* (WS 216–2001, before 2008) or *diagnostic criteria for dengue* (WS 216–2008, after 2008). The cases were classified as suspected, clinically diagnosed or laboratory-confirmed cases according to the diagnostic criteria. Generally, people were identified as suspected cases if he/she had more than two symptoms of acute onset fever, severe headache, orbital pain, myalgia, arthralgia, fatigue, (1) a history of travel in a dengue endemic area within 15 days before symptom onset, or cohabitation with an individual with confirmed dengue; or (2) a negative travel history, but with a rash or positive tourniquet test. Suspected cases with leucopenia or thrombocytopenia or serum IgM positivity were considered as clinically diagnosed cases. Laboratory-confirmed cases were the patient who were found to be positive for DENV RNA by real-time RT-PCR or for nonstructural protein 1(NS1) in acute-phase serum; those in which the virus was isolated from an acutely infected patient’s serum. The cases reported in this paper were laboratory-confirmed or clinically diagnosed cases, and all referenced as confirmed cases. More than 2 indigenous cases identified in a village/ community within 15 days was regarded as a dengue outbreak. Imported/indigenous cases were defined according to the epidemiological history. Cases were recorded as imported if he/she got the infection in places other than Zhejiang province. All laboratory testing were conducted in local county/city CDC. All dengue cases are required to be recorded in the National Notifiable Disease Surveillance System (NNDSS) within 24 hours after diagnosis, as required by the Law of the People’s Republic of China on Prevention and Treatment of Infectious Diseases. A standard questionnaire, which includes general individual information, progression of disease and treatment, symptoms, physical examination, laboratory test, contact and visiting history, was used to interview dengue cases or their relatives by staff from the local CDC. The data about symptoms, physical examination and visiting history were not available for all the cases. This is the limitation in this study.

### Study site

Zhejiang Province is located on the eastern coast of China. There are 91 counties and 11 prefecture level cities. It has a humid subtropical climate with four distinct seasons. Spring, from March to May, is a rainy season with changeable weather. Summer usually starts from June to September and is hot, rainy, and humid. Fall is kindly warm, sunny and relatively dry. Winter is short but cold except in the far south. The average annual temperature is around 15 to 19°C and annual precipitation is about 1,000 to 1,900 mm. There is plenty of rainfall in early summer, and the area directly threatened by typhoons forming in the Pacific in summer and early fall every year. Aedes aegypti mosquito have not been identified in any part of Zhejiang Province until now, but there is abundance of aedes albopictus in the summer and fall [10]. As one of the most developed provinces in China, personnel exchanges with abroad (including dengue epidemic area) is frequent in Zhejiang.

### Statistical methods

EpiData software version 3.1 (EpiData Association, Odense,Denmark) was used to establish a database. The epidemiological and clinical characteristics of dengue cases were summarized through frequencies for categorical variables and the median or mean for quantitative variables. Continuous data were compared using the Student’s t test or the Wilcoxon rank-sum test. Categorical variables were analyzed with the Chi-square or Fisher exact tests. The strength of association was evaluated with an odds ratio (OR) and its 95% confidence interval (CI). The relationship between the age and symptoms was explored with univariate logistics regression model, and age was transformed as ordinal variable. A significant difference was noted if *P* < 0.05. WPS Office 2016 (Kingsoft software service co. LTD., Beijing, China) and SPSS software version 17.0 (SPSS Inc., Chicago, IL, USA) were used for all the descriptive and statistical analyses.

SaTScan 9.1.1 was used to detect the space-time clusters of dengue and verify whether the clustering was caused by random variation or not. Circular scan windows and discrete Poisson models were used for scanning the high-rates area with monthly units. Likelihood ratio tests were evaluated to determine the significance of identified clusters and *P*-values were obtained through Monte Carlo simulation after 999 replications. The null hypothesis of a spatiotemporally random distribution was rejected when the *P*-value was < 0.05. The window with the maximum likelihood is defined as the primary cluster, that is, the cluster least likely to be due to chance. Other clusters with statistically significant log-likelihood ratios (*LLR*) were defined as the secondary potential clusters. The map of Zhejiang province was freely downloaded from National Earth System Science Data Sharing Infrastructure (http://www.geodata.cn/). ArcGIS (version 10.0.0, ESRI Inc., Redlands, CA, USA) software was used for the geographical-distribution-plotting.

## Results

### Demographic characteristics

**Fig 1:**
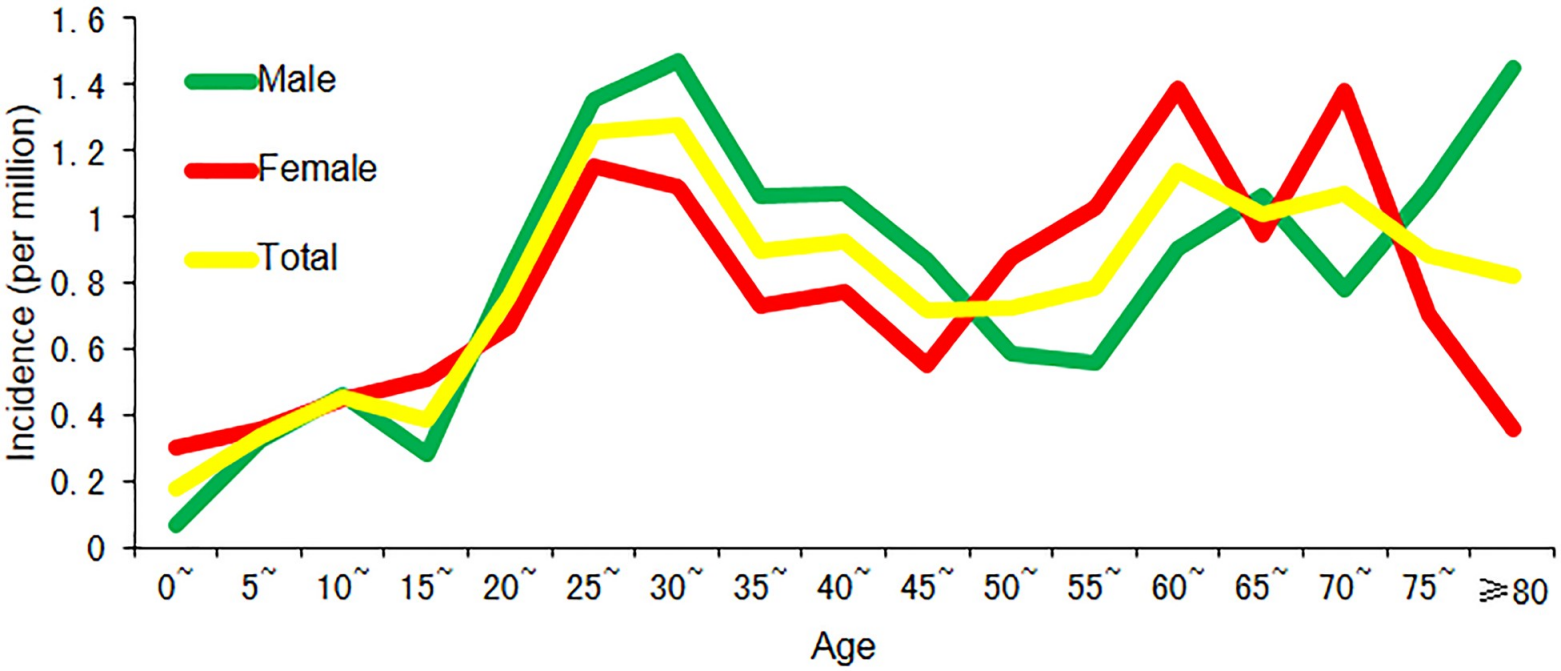
The dengue incidence for people at different age in Zhejiang Province.

A total of 529 dengue cases were reported in Zhejiang Province from 2005 to 2016, with 235 (44.4%) imported cases and 294 (55.6%) indigenous cases (Table 1). The annualized average incidence was 0.81 per million, with 0.82 per million for male and 0.80 per million for female. The overall male-to-female sex ratio was 1.09:1, and the gender distribution was significantly different between imported and indigenous cases with more male in imported cases (male-to-female sex ratio:1.80 vs 0.74, χ^*2*^ = 24.73, *P* <0.001).The overall mean age was 42 years (range: 1 to 96 years), and indigenous cases were significantly older than imported ones (47 years vs 35 years, *t* = -8.60, *P*<0.001). The incidence was highest in 30~35 age population (1.28 per million) as a whole, followed by 25~30 age group (Fig 1). Farmer, businessman and worker were the three most frequently reported occupations in dengue cases. The occupation distribution was also significantly different between imported and indigenous cases (χ^*2*^ = 188.60, *P* <0.001), with businessman as the most frequently reported occupation for imported cases and farmer for indigenous.

**Table 1:** The characteristic of dengue cases in Zhejiang Province from 2005 to 2016.

Characteristic		Imported	Indigenous	Total
**Age(years), mean(95%CI)**		35 (34, 37)	47 (45, 50)	42 (40,44)
**Gender, n (%)**	**Male**	151(64.3%)	125(42.5%)	276(52.2%)
	**Female**	84(35.7%)	169(57.5%)	253(47.8%)
**Occupation, n (%)**	**Farmer**	19(8.09%)	173(58.8%)	192(36.3%)
	**Businessman**	77(32.8%)	13(4.42%)	90(17.0%)
	**Worker**	37(15.7%)	30(10.2%)	67(12.7%)
	**Student**	20(8.51%)	22(7.48%)	42(7.94%)
	**Housework or unemployment**	16(6.81%)	24(8.16%)	40(7.56%)
	**Cadre or clerk**	28(11.9%)	3(1.02%)	31(5.86%)
	**Retiree**	8(3.40%)	6(2.04%)	14(2.65%)
	**Others**	30(12.8%)	23(7.82%)	53(10.0%)
**Median time interval (days), (inter-quartile range)**	**between onset and first visit**	1(0, 2)	0(0, 1)	1(0, 2)
	**between onset and confirmation**	6(4, 8)	7(4, 14)	6(4, 10)
	**between first visit to a health facility and confirmation**	6(4, 8)	7(4, 10)	6(4, 8)

### Spatial and temporal distribution characteristic

**Fig 2:**
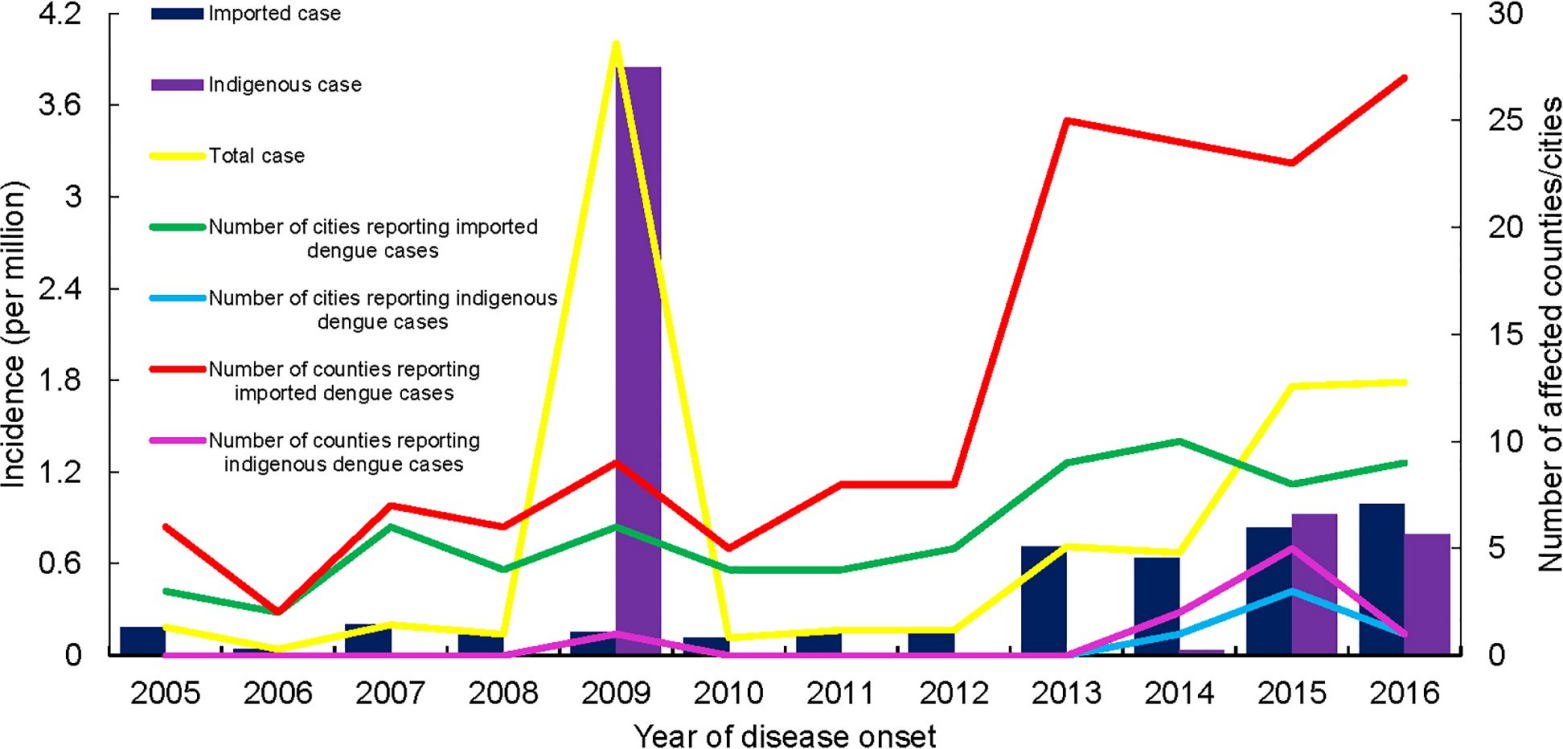
The incidence of dengue cases and the numbers of affected areas in Zhejiang Province from 2005 to 2016.

**Fig 3:**
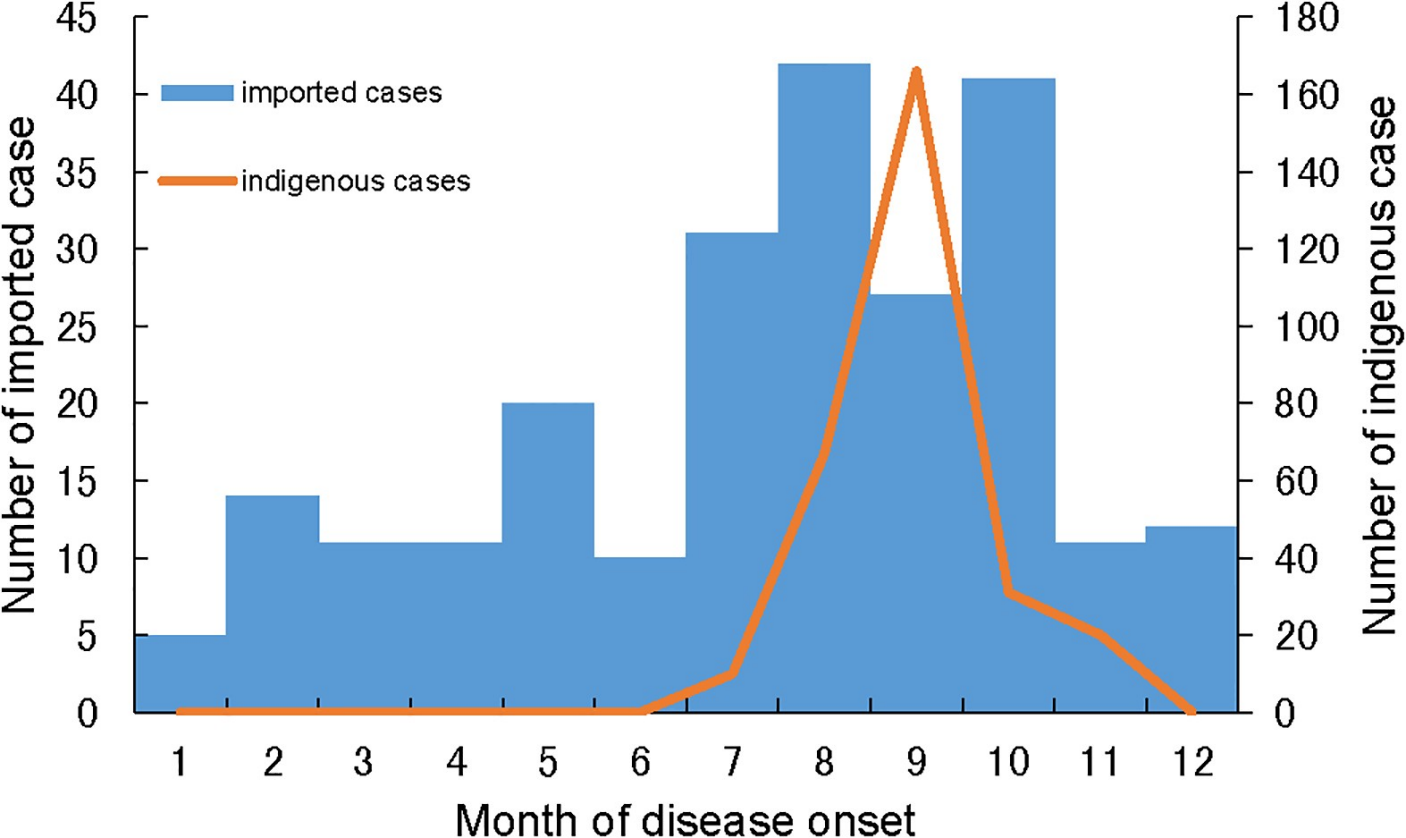
The monthly distribution of imported and indigenous dengue cases in Zhejiang Province from 2005 to 2016.

There were overall increases in the incidence of dengue cases and the number of counties reporting imported cases since 2011 (Fig 2). Indigenous cases were reported every year since 2014, which was rare in Zhejiang before then. The majority (70.7%) of notified cases during 2005 and 2016 occurred between the months of August and October (Fig 3). Imported cases were reported all year round, with a peak (60% of all notifications) between July and October. Indigenous cases were only reported between July and November, with 56.5% of indigenous cases reported during September.

**Fig 4:**
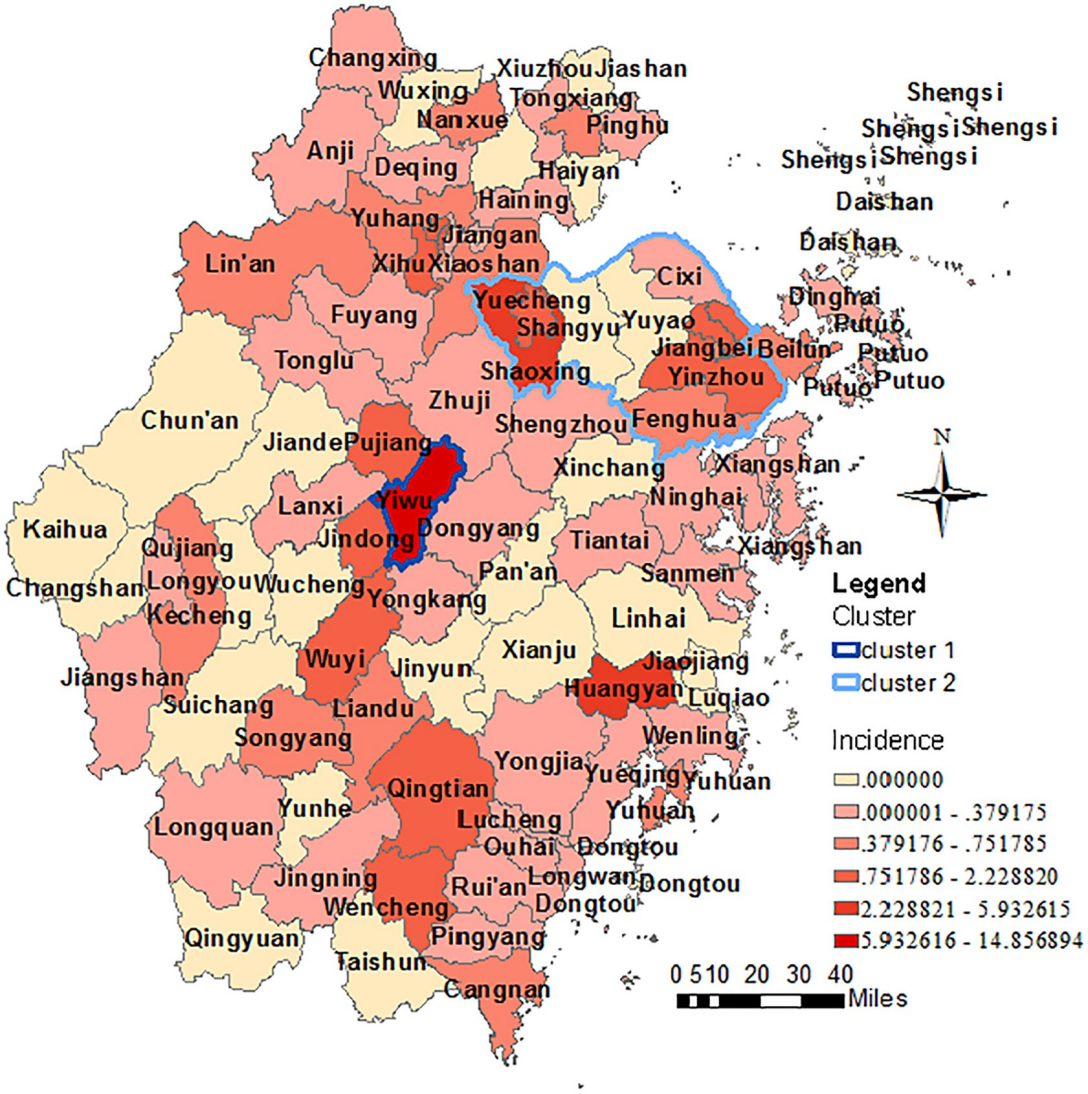
The geographic distribution and space-time cluster of dengue cases in Zhejiang Province from 2005 to 2016.

All 11 prefecture-level cities in Zhejiang Province reported dengue cases between 2005 and 2016, with the highest annualized average incidence reported in Jinghua(3.62 per million) and lowest in Zhoushan(0.14 per million, Fig 4). Indigenous cases were only diagnosed in Jianghua (2009), Wenzhou (2014), Shaoxing (2015), Taizhou (2016), Ningbo (2016) and Hangzhou (2016). Outbreaks were confirmed in Jianghua, Shaoxing and Taizhou. In total, 65 of 91 (71.4%) counties reported dengue cases during this period (Fig 4). 220 cases (41.6%) were reported in Yiwu, which made it as the county with the most cases. Indigenous cases were reported in 9/91 counties, with three counties accounting for 95.9% of all notified indigenous cases (Yiwu: 66.9%; Huangyan: 15.0%; Keqiao [previously called Shaoxing]: 13.9%). Dengue outbreaks were also confirmed in those three counties. Two statistically significant clusters were confirmed (Fig 4). Yiwu was recognized as the primary cluster (*P* <0.001) during the period from August to September in 2009. The secondary cluster (*P* <0.001) covered 11 counties (Haishu, Jiangdong, Jiangbei, Zhenhai, Yinzhou, Yuyao, Cixi, Fenghua, Yuecheng, keqiao, Shangyu) in the Northeast Zhejiang and span two months from August to September in 2015.

A total of 215 cases were infected abroad. Most of those cases were imported from Asia (85.1%), with 79.2% from Southeast Asia (S2 Table). Indonesia (26), Cambodia (25), Thailand (25), Philippines (19) and Malaysia (19) were the top five most frequently reported countries of origin. For the 20 cases imported from other provinces in China, Guangzhou(17) was the most commonly mentioned source province. The other three cases were imported from Yunnan(2) and Guangxi(1).

### Clinical presentation and medical-care-seeking

**Fig 5:**
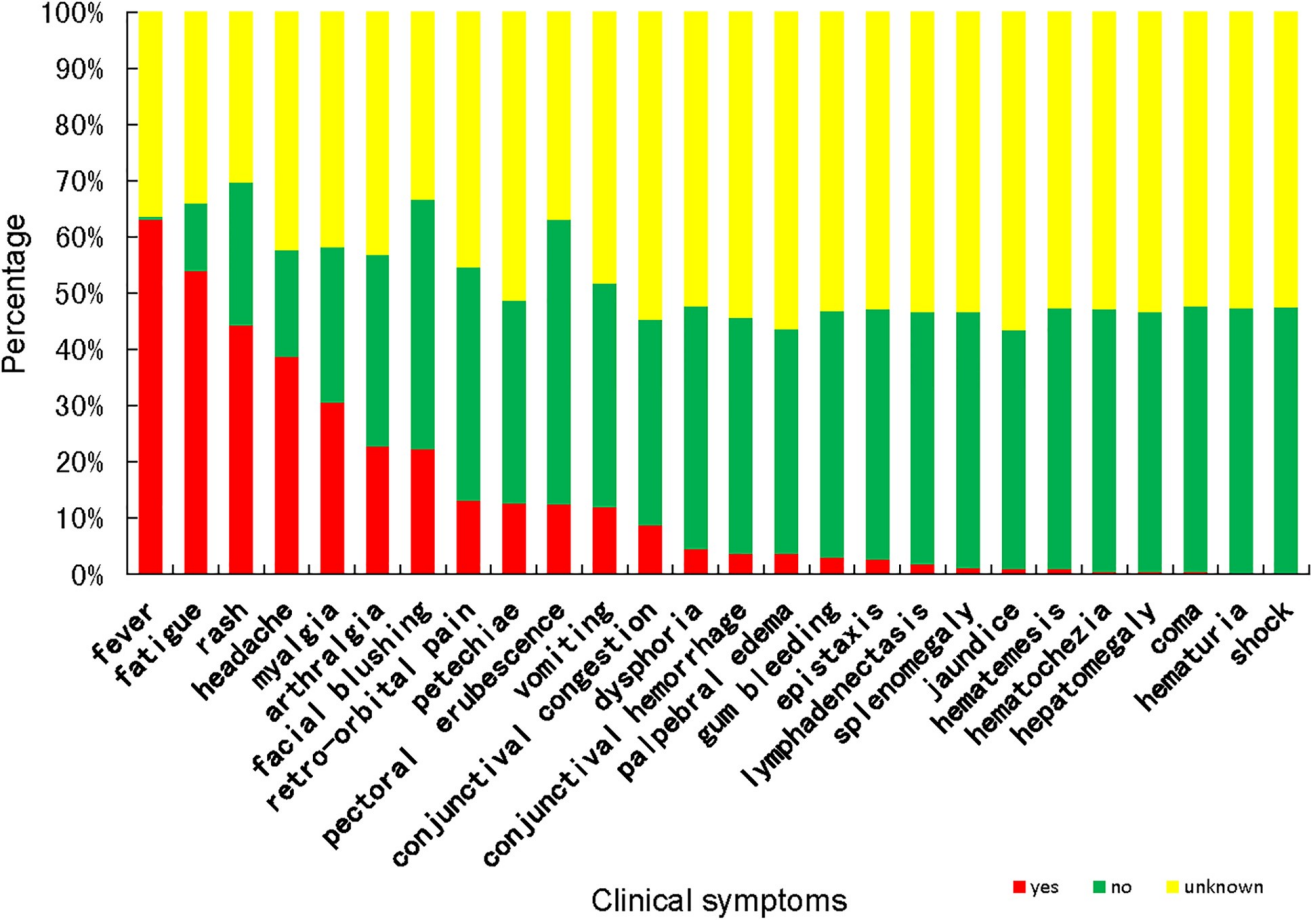
The clinical spectrum of dengue cases in Zhejiang Province from 2005 to 2016.

73.9% cases sought medical advice within two days after onset and were confirmed within 9 days after onset. 75.6% cases were confirmed as dengue within 8 days after their first visit. The time intervals between onset and confirmation (median 7 vs 6 days; Wilcoxon rank sum test *Z* = -2.40, *P* = 0.016), first visit and confirmation (median 7 vs 6 days; Wilcoxon rank sum test *Z* = -2.59, *P* = 0.009) for indigenous cases were significantly longer than those of imported ones. However, the time intervals between onset and first visit for indigenous cases was shorter than those of imported ones (median 0 vs 1 days; Wilcoxon rank sum test *Z* = -2.10, *P* = 0.036). Among the 339 cases who reported their visiting histories, 70.8% cases visited at least two different hospitals and 26.0% cases visited at least three different hospitals (S3 Table). The clinical spectrum of dengue cases were exhibited in Fig 5. No significant difference was noted in the distribution of symptoms between male and female, except for pectoral flushing and vomiting. Pectoral flushing was more frequently reported in male (19/153 vs 47/181, χ^*2*^ = 9.60, *P* = 0.002, *OR* = 0.845(0.761, 0.939)), while vomiting was more common for female (36/121 vs 28/153, χ^*2*^ = 4.95, *P* = 0.026, *OR* = 1.163 (1.013, 1.335)). The distribution of myalgia, arthralgia, bleeding gums, epistaxis and hemorrhagic manifestations were significantly different for different age groups (Table 2). Compared with cases young than 26, older cases had less chance to develop the symptoms of myalgia and arthralgia, and were more likely to have symptoms of bleeding gums, epistaxis and hemorrhagic manifestations.

**Table 2:** The relationship between the age and part of symptoms of dengue cases in Zhejiang Province during 2005 to 2016.

Symptoms	26–45 years old*				>45 years old*			
	n/N (%)	χ^*2*^	*P*	*OR*(95%CI)	n/N(%)	χ^2^	*P*	*OR*(95%CI)
**Myalgia**	92/154(59.7%)	7.95	0.005	0.404(0.215,0.759)	49/98(50%)	2.23	0.135	0.600(0.307,1.173)
**Arthralgia**	64/152(42.1%)	5.03	0.025	0.447(0.221,0.903)	44/96(45.8%)	6.36	0.012	0.384(0.183,0.808)
**Bleeding gums**	8/133(6.02%)	3.44	0.063	2.804(0.956, 8.227)	1/69(1.45%)	5.29	0.021	12.205(1.448,102.905)
**Epistaxis**	6/127(4.72%)	6.42	0.011	4.233(1.386,12.930)	0/69(0%)	0.00	0.997	_
**Hemorrhagic manifestations**^Θ^	60/138(43.5%)	2.72	0.099	1.755(0.899,3.426)	19/70(27.1%)	10.41	0.001	3.624(1.657,7.922)

* The dengue cases were divide into three groups according to their ages: 0–25 years old, 26–45 years old and >45 years old; cases aged 25 year or below was taken as reference.

^Θ^ Hemorrhagic manifestations: positive tourniquet test, petechiae, conjunctival hemorrhage, epistaxis, gum bleeding, hematemesis, hematochezia or hematuria.

## Discussion

Mirroring the Chinese mainland and world trends, Zhejiang witnesses an increase in case number and geographic expansion of dengue since 2012. The drivers behind this increase and expansion are thought to include globalization, increasing in mosquito breeding sites through rapid and often poorly planned urbanization, climate change, viral evolution and adaption, and deficiencies in water supply and garbage disposal [11]. The seasonality of dengue is similar to that in the whole country [12]. Imported cases were distributed all year around and peaked between July and October. Indigenous cases only had been reported in summer and fall and half of the cases occurred in September. The rainy and typhoon seasons in summer and fall created an abundance of water storage containers ideal for aedes mosquitoes breeding, and the warmer temperature accelerated viral transmission through increasing the infection rate and shortening the extrinsic incubation period [13–15], all of which may augment the mosquito population. Local transmission would be easily triggered by undetected dengue cases and asymptomatic infections in these seasons. Geographically, dengue cases were mostly distributed in the more developed north and central part of Zhejiang. And more than three quarters of imported cases were infected from Southeast Asia, which is similar to the situation in other provinces in China and Korea [16–18]. The first dengue outbreak after 1949 in Zhejiang Province was reported in Cixi, Ningbo prefecture level city, in 2004[19]. Then outbreaks were confirmed in Yiwu, Jinghua prefecture level city, in 2009, Keqiao, Shaoxing prefecture level city, in 2015 and Huangyan, Taizhou prefecture level city, in 2016. All three of these districts are well developed and characterized with high population mobility and frequent foreign trade. Yiwu is termed as the world's largest commodities market. Keqiao and Huangyan are famous for their textile industry, plastic and molding industry, respectively. The hotspots of these three outbreaks were all in the rural-urban continuum with or surrounded by numerous small-to-medium-sized private enterprises. The proportions of floating populations were high. The dwelling environments were also poor because of the higher population density, poor personal hygiene, casual littering and piling up idle water storage containers around buildings.

Unlike in epidemic areas where children and younger adults are high-risk populations, dengue cases in Zhejiang Province were older, with 67.7% cases between 25~60 years old [20]. A study conducted in Singapore, one of epidemic countries in Southeast Asia, showed that the age-weighted IgG prevalence of healthy adult residents is 50.8% (95%CI 49.4–52.3%) and the prevalence increases with age [21]. No related study had been conducted in China other than in Guangdong, the province most heavily affected by dengue. It showed that the overall sero-prevalence of dengue IgG antibody is 2.43% (range 0.28%–5.42%)[22]. All these suggest that the different age distribution of cases between Zhejiang and the dengue epidemic areas may be related to the fact that the human immunity of dengue virus is significantly low in non-endemic areas which makes the whole population generally susceptible. Compared to the indigenous cases, imported cases were younger with a higher percentage of males. It reflects the fact that younger male adults are more active, tend to be travel more frequently and thereby are under higher risk of dengue infection.

The median time interval between onset and confirmation for dengue case in Zhejiang Province was 6 days, and about three quarters of cases were confirmed in 9 days, which is similar to the situation reported in the mainland China as a whole [23]. For a small proportion of dengue-infected patients, the severe manifestations would develop during the course of the illness, usually day 4–6, at the time of fever clearance [24, 25]. This 48-hour period around the fever subsides has been classified as the “critical phase”, because patients require closer monitoring during this time. This study uncovered the fact that more than half of the dengue cases in Zhejiang Province could not have been confirmed in the “critical phase”, not to mention with close monitoring. The interval between onset and confirmation was significantly longer for indigenous cases. Besides that, the study conducted by Wang [23] indicated that age, occupation, place of residence, and date of onset would affect the time interval. The visiting history survey indicated that the delay in dengue diagnosis in Zhejiang was not caused by hesitation in medical-seeking. The clinical symptoms of dengue cases in Zhejiang Province are similar to those reported in other areas [22, 26]. Female, second-infection, infection with DENV II, children and elderly patient are recognized as factors that increase the risk of severe dengue fever[25, 26–28].Female dengue patients were found to be more likely to develop the symptoms of vomiting in this study and this symptom was confirmed as risk factor of severe dengue disease in an meta-analysis conducted by Zhang [29]. Besides that, our study also indicated that age might have an influence on the clinical symptoms. Studies conducted in India and Pakistan showed that dengue patients 40 years and older are significantly more likely to have longer hospital stay and die [30, 31].

In general, the trend of the dengue incidence is increasing and the geographical distribution is expanding in Zhejiang Province since 2011. Local transmission is recently reported more and more frequently. Although the symptoms of dengue are mild and the mortality is low, it has a profoundly negative socio-economic impact. Cases would seek medical advice in a timely manner and repeatedly after onset, but could not be confirmed in time. Dengue vaccines is available in several countries such as Mexico, and it is recommend by WHO that introduction is only considered in geographic settings where epidemiological data indicate a high burden of disease [32]. The vaccine is not recommended when seroprevalence is below 50% in the age group targeted for vaccination [32]. Considering no specific treatment for dengue and no vaccine available in Zhejiang Province, integrated vector management, timely diagnosis and disposition are the key preventive measures to stop local transmission and outbreak.

## Supporting information

S1 Table STROBE statement. (DOCX)

S2 Table The origin of the dengue cases imported from abroad in Zhejiang Province during 2005 to 2016. (DOCX)

S3 Table The distribution of the number of visited hospital for help in dengue cases in Zhejiang province during 2005 to 2016. (DOCX)
