# Supplementary material for: Epidemiological profile of dengue in Zhejiang Province, southeast China
Source: PLoS One. 2018 Dec 11;13(12):e0208810. doi: 10.1371/journal.pone.0208810 (PMC6289432; doi:10.1371/journal.pone.0208810)
Supplement: S2 Table — (DOCX) [file pone.0208810.s002.docx]

S2 Table. The origin of the dengue cases imported from abroad in Zhejiang Province during 2005 to 2016.

| source of infection | | number |
| --- | --- | --- |
| Asia | total | 183 |
|  | Southeast Asia | 145 |
|  | West Asia | 37 |
|  | South Asia | 1 |
| African | | 23 |
| America | | 8 |
| Oceania | | 1 |
