# Supplementary material for: Epidemiological profile of dengue in Zhejiang Province, southeast China
Source: PLoS One. 2018 Dec 11;13(12):e0208810. doi: 10.1371/journal.pone.0208810 (PMC6289432; doi:10.1371/journal.pone.0208810)
Supplement: S3 Table — (DOCX) [file pone.0208810.s003.docx]

S3 Table The distribution of the number of visited hospital for help in dengue cases in Zhejiang province during 2005 to 2016

| the number of visited hospital | N |
| --- | --- |
| 1 | 96 |
| 2 | 154 |
| ≥3 | 86 |
| unreported | 193 |
